# Mobile-phone-based e-diary derived patient reported outcomes: Association with clinical disease activity, psychological status and quality of life of patients with multiple sclerosis

**DOI:** 10.1371/journal.pone.0250647

**Published:** 2021-05-05

**Authors:** Daniel Golan, Smadar Sagiv, Lea Glass-Marmor, Ariel Miller

**Affiliations:** 1 Multiple Sclerosis Center & Department of Neurology, Lady Davis Carmel Medical Center, Haifa, Israel; 2 Rappaport Faculty of Medicine, Technion-Israel Institute of Technology, Haifa, Israel; Universita degli Studi di Napoli Federico II, ITALY

## Abstract

**Background:**

The applicability of mobile digital technology to promote clinical care of people with multiple sclerosis (pwMS) is gaining increased interest as part of the implementation of patient-centered approaches. We aimed at assessing adherence to a smartphone-based e-diary, which was designed to collect patient-reported outcomes (PROs). Secondary objectives were to evaluate the construct and predictive validity of e-diary derived PROs and to explore the various factors that were associated with changes in PROs over time.

**Materials and methods:**

In this observational cohort study patients downloaded an MS tailored e-diary into their personal smartphones. Report of PROs was enquired once monthly for a period of one year through a smartphone-based application, using previously validated tools. An e-diary derived bodily function summary score (eBF) was defined as the sum of scores depicting vision, limbs function, pain, bowl/ bladder dysfunction, pseudobulbar affect and spasticity. Multiple linear regression and analysis of covariance were used to determine the association between PROs, clinician-reported outcomes (ClinROs) of disease activity and quality of life (QoL). Regression coefficient analysis was used to compare the slope of change in eBF before and after a relapse.

**Results:**

97 pwMS downloaded the e-diary [Female: 64 (66%), EDSS 3.4±2.1]. 76 patients (78%) completed the 12-month study period. 53 patients (55%) submitted ≥75% of requested surveys. Anxiety was negatively associated with adherence to periodic PROs assessments by the e-diary. E-diary derived PROs were significantly correlated with corresponding functional system scores (0.38< r <0.8, P<0.001). eBF score significantly predicted QoL (β = -0.36, P = 0.001) while EDSS did not. Change in eBF score over time was independently associated with the occurrence of an MS relapse (F = 4.4, P = 0.04), anxiety (F = 6.4, P = 0.01) and depression (F = 5.1, P = 0.03). Individual regression slopes of eBF scores were significantly higher pre-relapse than post-relapse (3.0±3.3 vs. -0.8±2.0, P = 0.007).

**Conclusion:**

Adherence of pwMS to recording in an e-diary collecting PROs was high. Changes in e-diary derived PROs over time predict clinical MS relapses on the group level and thus carry the potential of usage in clinical research as well as for improved MS care in real world setting.

## Introduction

The advent of mobile electronic platforms opened up the possibility for interactive diaries that remind patients to enter data, prospectively collect information about patients’ symptoms while at their natural environment and present a comprehensive reflection of disease status from the patients’ perspective as part of the implementation of patient centric approach and participatory medicine [1–4]. As the everydayness of our lives becomes increasingly digitized, data generated outside of healthcare encounters holds promise to fill recognized gaps in real-world evidence [5].

Collection of patient-reported outcomes (PROs) by electronic diaries (e-diaries) may be suitable to supplement clinician-reported outcomes (ClinROs) for people with MS (pwMS) [6]. PROs have many potential uses, such as promoting communication between patients and physicians, assessing change in disease status, screening for unidentified symptoms as well as monitoring safety and efficacy of medications [7, 8]. Traditional physician derived endpoints, like the neurological examination and the expanded disability status scale (EDSS), do not capture the gamut of MS symptoms, such as pain, fatigue, depression and sleep disturbance. These are better depicted by PROs, which despite being subjective, have been shown to correlate with objective assessments [9–11], predict objective disease worsening [12] and have an important association with quality of life (QoL) [13]. Traditionally, PROs have been assessed using paper questionnaires but on-line administration of PROs gains popularity and has been shown to be equivalent [14]. In most assessments of currently available electronic tools related to medical use, the most prevalent functionalities were providing users with information/education, assisting users with their therapy adherence and helping users monitor the effect and possible side effects of their medications [15]. Despite the abundance of healthcare-related applications very little research has been undertaken to investigate the validity and efficacy of these tools as well as their potential risks [16]. A randomized clinical trial demonstrated that a mobile device application enhances reporting of adverse drug reactions by pwMS compared to traditional reporting by phone or e-mail [17]. A small study of an MS custom application designed to gather both PROs and objective performances (motor, cognitive and visual) reported high attrition among patients with visual as well as with subjective cognitive difficulties [18]. Still, other researchers have shown that the majority of pwMS use mobile phones regularly and are competent to use electronic patient relationship management systems [19]. It has also been reported that pwMS frequently use internet-based social media to gain knowledge about their disease and its management and are capable to distinguish reliable users, from those who post inaccurate information [20].

We developed an electronic diary (e-diary) smartphone application for longitudinal collection of PROs from pwMS. The potential added values of the smartphone application are: (1) to improve clinical practice by conveying summary scores of various disease related symptoms to clinicians. These scores and their change over time may facilitate discussion between patients and their caregivers regarding those symptoms and their management. (2) valid PRO summary scores can be used as efficacy endpoints in clinical trials of novel therapies. (3) electronic PRO measures can promote Patient-Powered Research Networks (PPRN) by collecting patient data with greater frequency than clinic visits, as already implemented by the Patient-Centered Outcomes Research Institute (PCORI) [21]. In this study we aimed at evaluating adherence to an electronic diary (e-diary) smartphone application for longitudinal collection of PROs from pwMS over 12 months and to describe predictors for adherence. Secondary objectives were to evaluate the construct and predictive validity of the e-diary derived PROs and to explore the various factors, disease related and psychological, that were associated with changes in PROs over time.

We premised good adherence to the tool and significant correlations between e-diary derived PROs and related clinician-reported outcomes. We hypothesized that changes in e-diary derived PROs over time would be associated with anxiety and depression but would nevertheless predict clinical MS relapses. We also hypothesized that e-diary derived PROs will be predictive of participants’ quality of life.

## Materials and methods

This was an observational cohort study. All participants were recruited at the multiple sclerosis clinic of Carmel medical center, Haifa, Israel. Patients were enrolled between February 2016 and January 2017. Data collection was continued until the last patient completed 12 months follow-up.

### Participants

97 pwMS according to the revised McDonald criteria were recruited for this study. Inclusion criteria were: age 18 to 70 years, able to browse the internet and use a smartphone, willing and able to give informed consent, EDSS ≤ 7. Patients with neurological conditions involving the central nervous system other than MS were excluded. Patients were offered to take part in the study consecutively, during their routine clinic visits. Of the patients approached, only five declined to participate. The study was approved by the institutional review board of the Lady Davis Carmel Medical Center, Haifa, Israel. IRB approval number: CMC-0065-13. All participants gave written informed consent.

### The e-diary application

An e-diary, tailored for pwMS, was developed. The e-diary is an internet smartphone application. Previously validated PROs questionnaires from multiple sources were computed into the e-diary application in order to cover the spectrum of MS related symptoms. The PROs that were used and data regarding their internal consistency, reliability and validity are elaborated in the S1 Table. Our intention in selecting the composition of PROs to be computed into the e-diary was to create a tool that could specifically grade MS symptoms in a comprehensive manner, with emphasis on symptoms frequency and intensity. PROs selection was in line with the intended purposes of the e-diary to enable patients to convey a comprehensive report of their symptoms in a timely and efficient manner, as well as a potential tool to collect PROs as efficacy end-point in therapeutic clinical trials.

PROs surveys were divided into two components, bodily function and mental. The bodily function survey included the impact of visual impairment scale, pain effects scale and bowel & bladder control scales from the MS quality of life inventory (MSQLI) [22]. Additionally included in the bodily function survey were the upper and lower extremity function short forms from the Neuro-QOL inventory [23], the numeric rating scale to measure spasticity [24] and the Center for Neurologic Study- Lability Scale (CNS-LS) to measure pseudo-bulbar affective lability [25].

The mental survey consisted of the mental health inventory, which measures anxiety, depression, positive affect and behavioral control, as well as the abbreviated modified fatigue impact scale, both from the MSQLI [22]. Also included in the mental survey were the abbreviated perceived deficits questionnaire [22], which measures subjective cognitive complaints, and the sleep disturbance short form [23].

Plots could be produced by the application, which presented PROs summary scores of each domain by time from study entry. These plots enabled physician and patients to gain insight into the various symptoms scores and their trajectory over time. The application also sent reminders to take disease modifying drugs (DMDs) and asked users to report their DMDs intake. Monitoring of adherence to DMDs by this e-diary was reported elsewhere [26].

### Study measures and procedures

Patients were clinically evaluated at baseline, after 6 months and by the end of a 12 month follow up period. On each visit relapses were recorded, and a detailed neurological examination was performed and quantified according to the neurostaus definitions. EDSS score as well as pyramidal, cerebellar, sensory, brainstem bowel & bladder and visual system scores were obtained [27].

At baseline, QoL was assessed using the abbreviated version of the generic world health organization quality of life questionnaire (WHOQOL-BREF) [28]. This QoL measure has a broad scope, including ‘physical health and autonomy’, ‘psychological health’, ‘social relationships’ and ‘environmental aspects’. The total score is between 0 (worst health) to 20 (best health). The WHOQOL-BREF showed satisfactory levels of internal consistency with Cronbach’s alpha between 0.63 and 0.81among pwMS [29].

Anxiety and depression were evaluated at baseline with the Hospital Anxiety and Depression Scales (HADS). A cut-point of ≥11 indicates clinically meaningful symptoms of anxiety and depression [30]. Cognitive function at baseline was screened using the Symbol Digit Modalities Test (SDMT) [31]. Raw scores were converted to age adjusted z-scores, using normative data [32]. Baseline evaluations of QoL, anxiety, depression and cognition were assessed using traditional paper and pencil questionnaires.

Participants were asked to submit a comprehensive monthly PROs report by means of the e-diary application. The e-diary reminded participants to submit the monthly report. In addition, patients could submit additional reports any time, at their discretion. A summary e-diary derived bodily function score (eBF) was computed as the sum scores of the ’bodily function’ components, namely the sum of visual impairment, lower and upper extremity, pain, bowel & bladder, pseudobulbar and spasticity sub scores (S1 Table).

Construct validity of e-diary derived PROs was assessed by looking for correlations between subjective symptom-specific measures and physician determined systems scores of the corresponding domains [27]. Predictive validity of e-diary derived PROs was estimated by looking for independent association with QoL and by looking for an association between change in e-diary derived PROs over time and MS relapse activity.

### Statistical analysis

**Fig 1:**
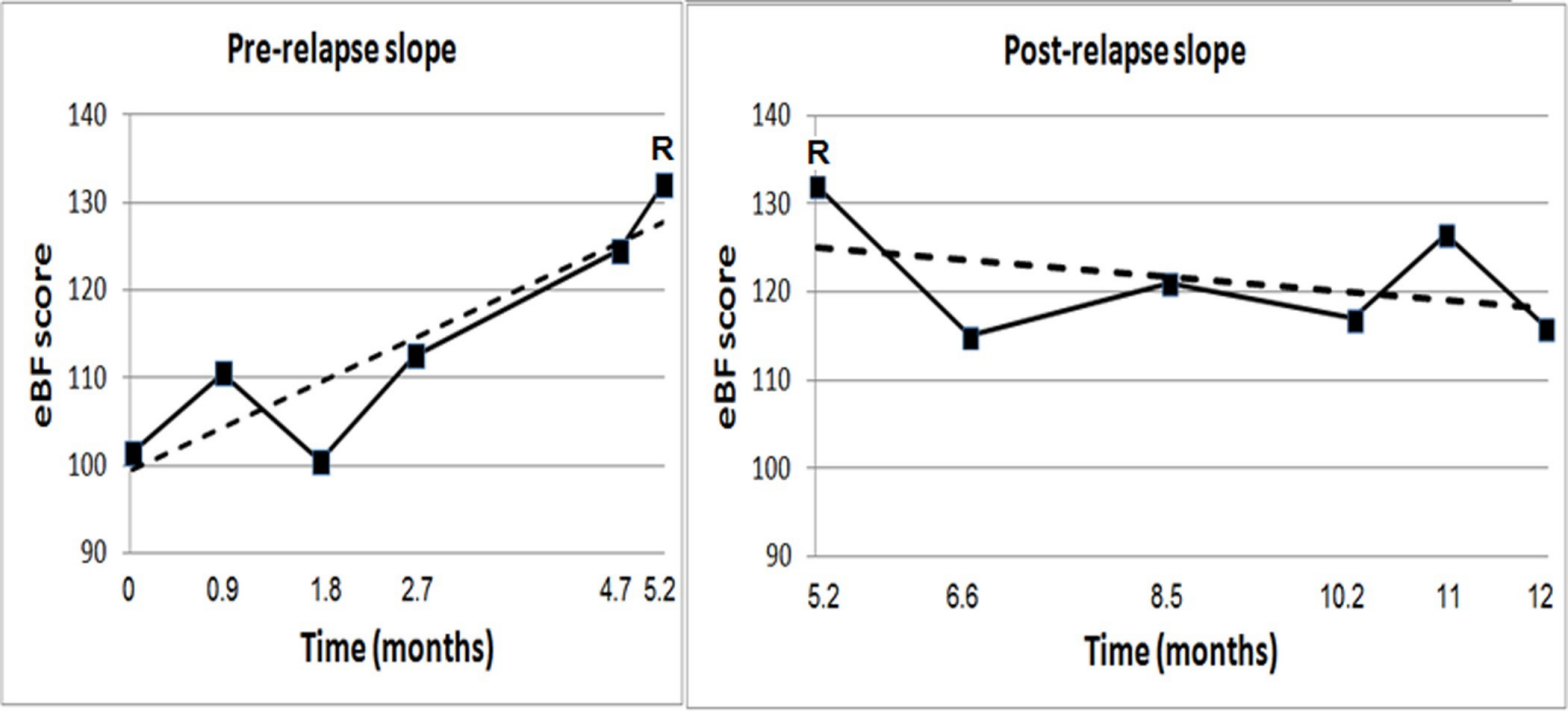
Pre- and post-relapse regression slopes: Illustration for one patient. The sum scores of e-diary derived bodily function PROs by time since study entry from a single participant are shown. This patient had an on-study relapse about 5 months after enrolment to the study (marked by the letter ’R’). The trend of change in bodily function PROs score is depicted by the dashed line, which represents the individual regression slope [33]. The pre-relapse regression slope (left panel) is positive, indicating worsening, while the post-relapse regression slope (right panel) is negative, indicating improvement.

Data analysis was performed using SPSS, version 23.0 (IBM Corp., Armonk, N.Y., USA). Data distribution was inspected for normality. Continuous variables were analyzed using between-groups t-test. Categorical variables were analyzed with chi-square or Fisher’s exact tests. Correlation coefficients are Pearson’s r or Spearman’s rho, according to data distribution. Linear regression modeling was used to assess the independent contribution of variables that were significantly associated with QoL in the univariate correlation analyses. Maximal change in bodily function PROs was computed for each participant as the difference between the maximal and minimal e-diary derived bodily function score (maximal eBF score minus minimal eBF score). Analysis of covariance (ANCOVA) was used to assess the independent contribution of anxiety, depression and clinical MS relapses to maximal change in bodily function PROs over time. Regression coefficient analysis and individual regression slopes were used to assess the trend of change in individual eBF scores over time [33]. For participants with an on-study relapse, pre relapse regression slopes were determined between baseline and relapse date. Post relapse regression slopes were determined between relapse date and 6 months post relapse or end of follow up, whichever obtained first (Fig 1). Pre relapse regression slopes were compared to post relapse regression slopes using paired t-test. Pre relapse regression slopes were also compared to whole study regression slopes of patients who did not relapse during the study (baseline to end) using independent groups t-test. Descriptive statistics are reported as mean ± standard deviation or frequency (%). Sample size considerations are detailed in the (S1 File).

## Results

### Participants and adherence to the e-diary

**Fig 2:**
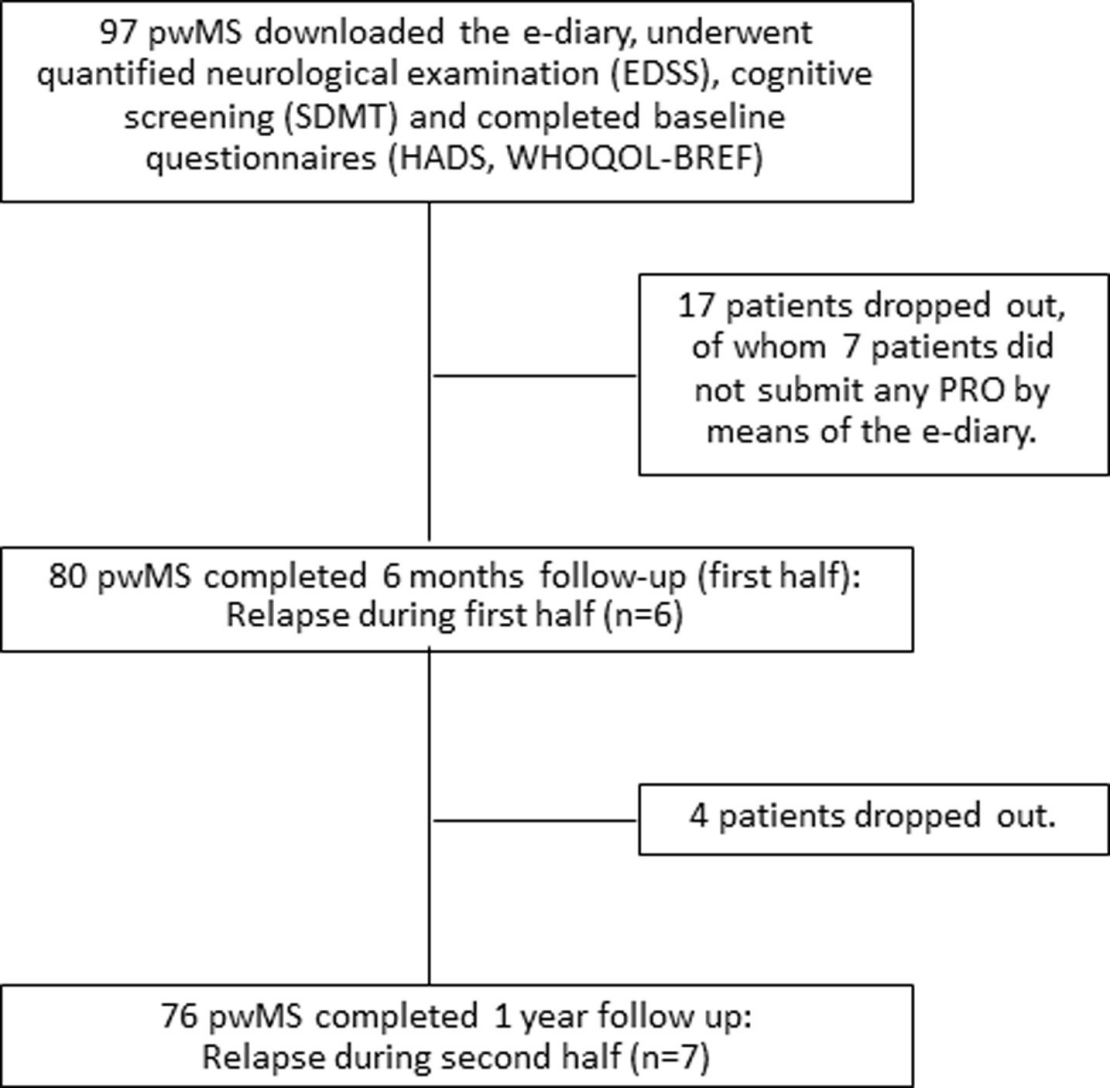
Study flow chart. 21 patients dropped out before 12 months of intended follow up. EDSS = Expanded Disability Status Scale. SDMT = Symbol Digit Modalities Test. HADS = Hospital Anxiety and Depression Scale. WHOQOL-BREF = World health organization quality of life questionnaire. pwMS = people with multiple sclerosis.

Demographic and clinical characteristics of the 97 patients who downloaded the e-diary application are given in Table 1. Study flow chart is shown in Fig 2. Drop out was defined as complete cessation of e-diary activity or non-show to clinical follow up. Patients who remained partially cooperative in the study were not regarded as dropouts. 17 patients dropped out before 6 months of follow up and additional 4 dropped out before 12 months of intended follow up. Reasons for discontinuation were: anxiety provoked by the PROs surveys (n = 5), internet or smart-phone malfunction (n = 4), did not attend clinic visits (n = 4), could not learn how to use the application (n = 3), time consuming / not interested (n = 3), pregnancy (n = 1), language difficulties (n = 1) and loss to follow up (n = 1).

**Table 1:** Patients’ characteristics.^a^

**N**	97
**Age**	40.4±11 (18–63)
**EDSS**	3.4±2.1 (0–7)
**Disease duration (From diagnosis, years)**	9.1±8.0
**Female**	64 (66%)
**Primary language**^b^	Hebrew 84 (87%)
	Arabic 13 (13%)
**Education**	Academic 59 (61%)
	Technical 16 (17%)
	High school 22 (22%)
**MS type**	RRMS 90 (93%)
	SPMS 6 (6%)
	PPMS 1 (1%)
**Active disease at enrolment (Relapse or MRI new lesion in previous year)**	37 (38%)
**Cognitive impairment according to SDMT**^c^	31 (32%)
**DMT at baseline**	Fingolimod 39 (40%)
	Dimethyl fumarate 24 (25%)
	Interferon beta 12 (13%)
	Glatiramer acetate 9 (9%)
	Teriflunomide 6 (6%)
	Natalizumab 4 (4%)
	None 3(3%)

a. Summary statistics are mean ± standard deviateon.

b. E-diary application questionnaires were in Hebrew.

c. SDMT = Symbol Digit Modalities Test. PwMS with age-adjusted z-score of -1.5 or lower were defined as cognitively impaired [34].

**Fig 3:**
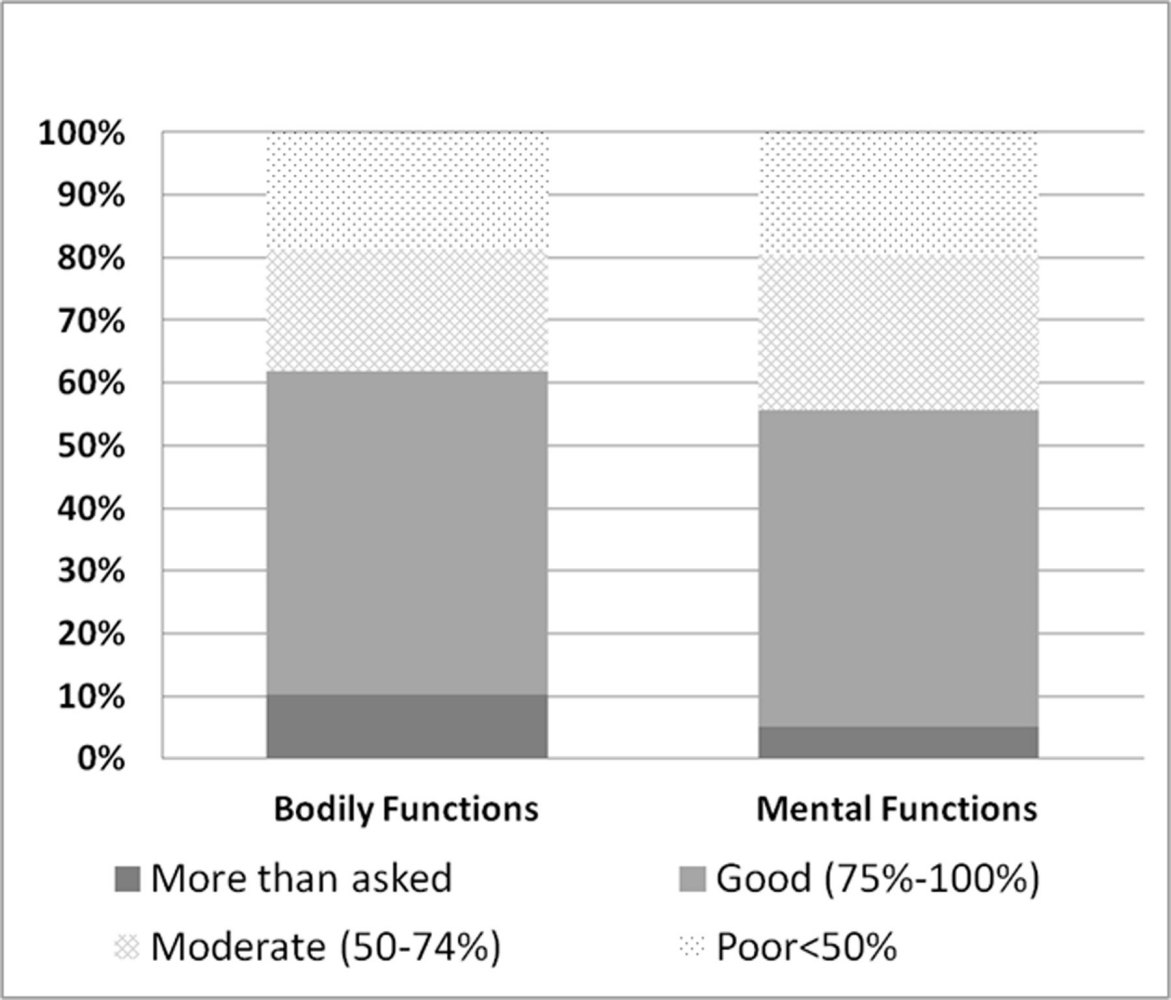
Adherence to PROs collection by means of the e-diary. Patients were asked to submit a bodily function report and a mental function report at least once a month. 80% of participants submitted at least 50% of the required PROs reports, while about 60% of participants submitted at least 75% of the required surveys. The adherence to bodily function reporting was slightly higher compared to the adherence to the mental function survey.

Adherence to PROs collection by means of the e-diary is presented in Fig 3. Patients were asked to submit a bodily function report and a mental function report at least once a month. 80% of participants submitted at least 50% of the required PROs reports, while about 60% of participants submitted at least 75% of the required surveys. The adherence to bodily function reporting was slightly higher compared to the adherence to the mental function survey.

Predictors of adherence to PROs surveys are presented in Table 2. Female patients were more adherent than male patients. Anxiety was associated with poor adherence to PROs surveys.

**Table 2:** Predictors of adherence to patient reported outcomes collection by the e-diary.

*Characteristic*	*E-diary high adherence ≥75% submission rate (all surveys)*	*E-diary moderate/poor adherence*	*P value* ^a^
	*N = 53*	*N = 44*	
***Age***	39.5±10.9	41.3±11.2	0.4
***Gender***	F = 40 (76%)	F = 24 (55%)	0.03
***EDSS (baseline)*** ^***b***^	3.3±2.1	3.5±2.1	0.6
***Disease duration* (From diagnosis, years)**	7.9±7.4	10.4±8.7	0.2
***Baseline active disease*** ^***c***^	16 (30%)	21 (48%)	0.08
***Visual system score ≥ 2*** ^***d***^	9 (17%)	11 (25%)	0.3
***SDMT (baseline)*** ^***e***^	45.9±13	41.1±11.8	0.06
***Baseline depression (HADS)*** ^***f***^	6.6±4.7	7.0±3.5	0.35
***Baseline anxiety (HADS)*** ^***f***^	7.8±5.0	10.1±5.2	0.03
***Employed***	27 (51%)	19 (43%)	0.4
***Primary language other than Hebrew*** ^***g***^	8 (15%)	5 (11%)	0.3

a. P values for continuous variables are from an independent group t-test. P values for categorical variables are from a chi square test.

b. EDSS = Expanded Disability Status Scale.

c. Active disease at baseline was defined as a clinical relapse, a new MRI lesion or an enhancing MRI lesion in the year prior to recruitment.

d. According to ’neurostatus’ visual system score, at baseline [27].

e. SDMT = Symbol Digit Modalities Test [31].

f. HADS = Hospital Anxiety and Depression Scale [30].

g. E-diary application questionnaires were in Hebrew.

### Construct validity of e-diary derived PROs

Correlations between e-diary derived PROs and quantified neurological examination scores at baseline are given in Table 3. Strong correlation was found between the sum of bodily function scores (eBF) and EDSS. Strong correlations were also found between the pyramidal system score and PROs of spasticity, lower and upper limb functions. Moderate correlations were found between sensory and visual system scores and corresponding PROs of pain and visual functions. Subjective PROs of cognitive difficulties and limb functions were weakly correlated with SDMT and cerebellar system scores, respectively. E-diary derived depression and anxiety scores were strongly associated with corresponding HADS-D and HADS-A scores (Pearson r = 0.68 for depression and 0.72 for anxiety, P value <0.001).

**Table 3:** Correlations between e-diary derived patient reported outcomes and quantified neurological examination scores at baseline.^a^^,^^b^

*PRO Ediary* ^*c*^	*Quantified neurological examination* ^*a*^	*Correlation Coefficient* ^*d*^^,^^*e*^	*P value*
Sum ’Bodily function’	EDSS ^f^	0.77	< .001
Spasticity	Pyramidal	0.71	< .001
Upper limb	Pyramidal	0.69	< .001
	Cerebellar	0.35	.01
Lower limb	Pyramidal	0.8	< .001
	Cerebellar	0.38	< .001
Pain	Sensory	0.5	< .001
Visual	Visual	0.5	< .001
Cognitive difficulties	SDMT ^g^	0.33	.002

a. According to ’neurostatus’ functional system scores, at baseline [27].

b. Data was available from 90 patients.

c. PRO = Patient reported outcome.

d. Correlation coefficients of visual and upper limb patient reported outcomes are Spearman’s rho, due to non-normal distribution. All other correlation coefficients are Pearson’s r.

e. By convention, correlation coefficients of 0.7, 0.5 and 0.3 represent strong, medium and weak correlation, respectively.

f. EDSS = Expanded Disability Status Scale.

g. SDMT = Symbol Digit Modalities Test [31].

### Predictive validity of e-diary derived PROs: Association with QoL

Correlations between various disease characteristics and QoL at baseline, as measured by the generic world health organization quality of life questionnaire (WHOQOL-BREF) are given in Table 4. On univariate analysis, the sum of bodily function scores (eBF), fatigue, anxiety, subjective cognitive difficulties and sleep were strongly correlated with QoL. EDSS, depression and SDMT were moderately correlated with QoL at baseline. Multivariate analysis showed that only the sum of bodily function PRO scores (eBF), depression and anxiety were independently associated with QoL. Objective/ physician derived measurements, such as EDSS and SDMT were not independently associated with QoL in this study.

**Table 4:** Quality of life predictors at baseline.

Variable	Univariate analysis			Multiple linear regression^a^	
	N	Pearson r	P value	Standardized beta	P value
**Sum of bodily function PROs** ^**b**^ **(e-diary)**	**90**	**-0.78**	**<0.001**	**- 0.37**	**<0.001**
**Depression (HADS)** ^**c**^	**97**	**-0.63**	**<0.001**	**- 0.32**	**<0.001**
**Anxiety (HADS)** ^**c**^	**97**	**-0.74**	**<0.001**	**- 0.14**	**0.05**
**Fatigue (e-diary)**	87	-0.78	<0.001	- 0.17	0.11
**Subjective cognitive difficulties (e-diary)**	87	-0.72	<0.001	- 0.10	0.26
**EDSS** ^**d**^	97	-0.48	<0.001	0.07	0.45
**SDMT** ^**e**^ **(z score)**	96	0.42	<0.001	0.02	0.76
**Sleep (e-diary)**	87	-0.67	<0.001	0.01	0.89

a. 81% of the variance in quality of life was explained by the model.

b. PROs = Patient reported outcomes.

c. HADS = Hospital Anxiety and Depression Scale [30].

d. EDSS = Expanded Disability Status Scale.

e. SDMT = Symbol Digit Modalities Test [31].

### Predictive validity of e-diary derived PROs: Association with change in disease status

**Fig 4:**
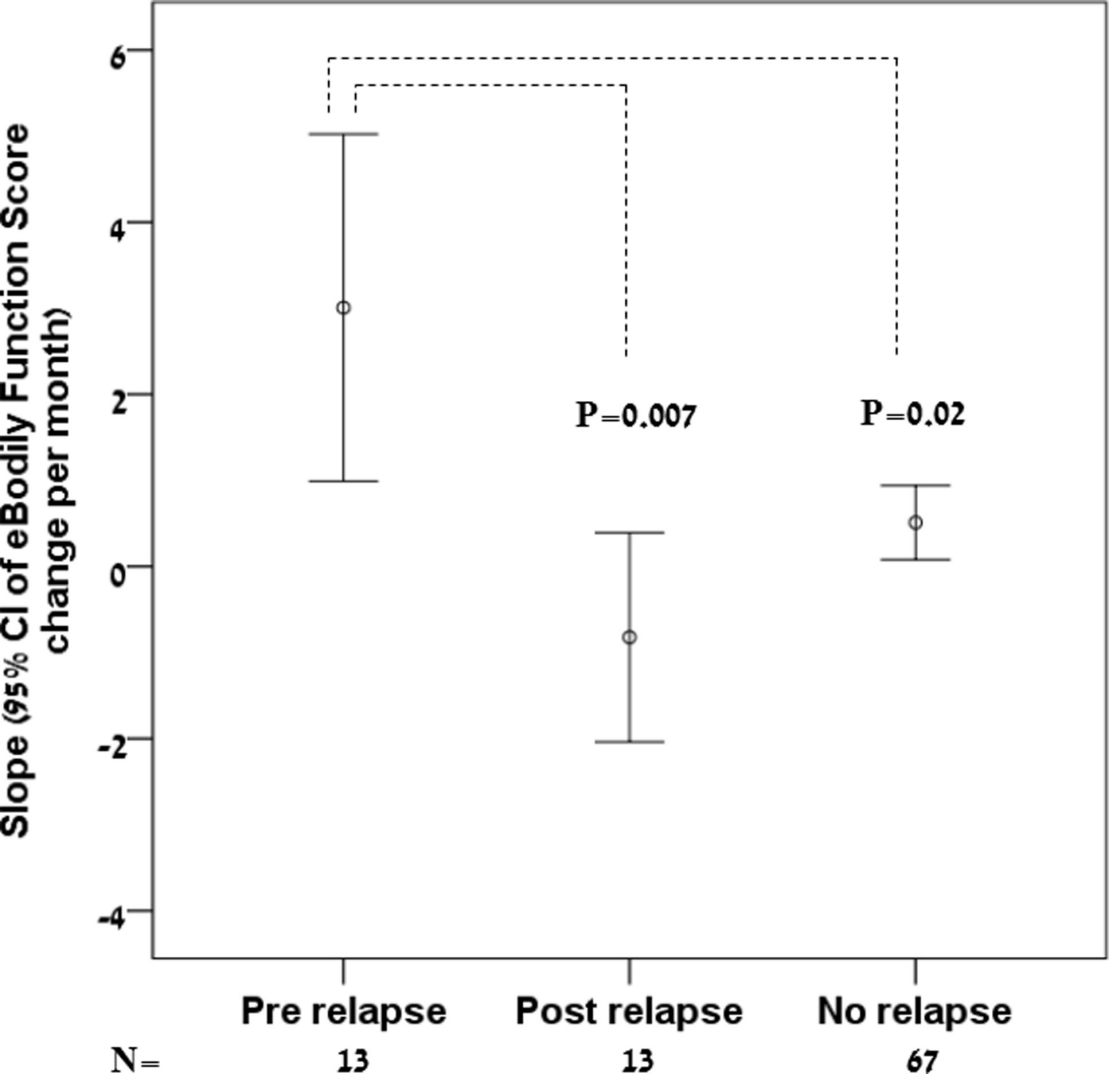
Distribution of individual regression slopes of the sum of bodily function scores (eBF). 13 patients had a relapse during the study. The average pre-relapse slopes were positive (indicating worsening), while post-relapse slopes were negative on average (indicating improvement). Individual regression slopes of patients who did not relapse during the study were close to zero. P value for the comparison between pre-relapse and post-relapse regression slopes is from a paired t-test. P value for the comparison between pre-relapse regression slopes and regression slopes of patients without clinical MS relapses is from an independent group t-test.

The distribution of individual regression slopes of the sum of bodily function scores (eBF) are presented in Fig 4. 13 patients had a relapse during the study. The average pre relapse slopes were positive (indicating worsening), while post relapse slopes were negative on average (indicating improvement). Individual regression slopes of patients who did not relapse during the study were close to zero. Pre-relapse regression slopes were significantly higher than both post-relapse regression slopes, and regression slopes of patients who did not experience an MS exacerbation during the study.

The independent effect of predictors of maximal change over time in e-diary derived bodily function PROs are given in Table 5. Depression and anxiety were independently associated with change in bodily function PROs over time. On study MS relapse was also an independent predictor of change in e-diary derived bodily function PROs. There were insignificant interactions between depression and on-study relapse as well as between anxiety and on-study relapse in association with maximal change in e-diary derived bodily function patient reported outcomes.

**Table 5:** Predictors of maximal change over time in e-diary derived bodily function patient reported outcomes.

Variable	F-Value^a^	P-value^a^
MS relapse during the study	4.4	0.04
Depression (baseline HADS^b^)	5.1	0.03
Anxiety (baseline HADS ^b^)	6.4	0.01

a. P values are from an analysis of covariance (ANCOVA) on data from 90 patients who submitted multiple bodily function reports using the e-diary.

b. HADS = Hospital Anxiety and Depression Scale [30].

## Discussion

In this study we show that pwMS can adhere to continuous collection of PROs by a smartphone-based e-diary. These PROs represent disease aspects with a significant and independent association with patients’ QoL. E-diary derived bodily function PROs were associated, tough not identical, with their corresponding domains from the quantified neurological examination, in support of their construct validity. Change in bodily function PROs over time on the group level was independently associated with clinical MS relapses, anxiety and depression.

The overall adherence to the e-diary as a tool for longitudinal PROs evaluation was acceptable. About 80% of participants submitted at least 50% of the required surveys, meaning that for 80% of patients at least one PROs report was available every 2 months, which is more often than the usual frequency of clinic visits. Thus, the e-diary carries the potential of tapping patients’ status in between clinic visits. This finding is in line with previous reports of higher adherence to e-diaries, when compared to paper diaries, partly due to compliance-enhancing features as alarms and reminders [35–37]. While it has been reported that male and younger patients had more favorable perceptions of PROs collection [7], we found better adherence to PROs surveys among female pwMS. Age was not significantly influencing adherence to PROs collection in this study, however most of our patients were young (Mean age 40.4). E-diary application questionnaires were in Hebrew. Although one patient dropped out due to language difficulties, primary language other than Hebrew was not associated with adherence to PROs collection by means of the e-diary.

Anxiety turned out to be a major cause of both complete drop-out and decreased adherence to PROs surveys. Patients reported that the PROs questionnaires exposed them to anxiety provoking awareness of MS related symptoms that they did not yet experience. Interestingly, the same observation was made with an e-diary for patients with headache. Endorsing higher daily anxiety was associated with lower odds of complete reporting [38]. Future e-diaries for pwMS should be personalized. A general question about the relevance of a symptom cluster to the respondent should be introduced first and detailed specific questions should follow only if necessary.

The bodily function component of the e-diary is based upon subjective measures, which were originally designed to capture the impact of neurological symptoms upon QoL, such as the MS quality of life inventory (MSQLI) [22] and the Neuro-QOL inventory [23]. The correlations between patients and physicians scores varied for different symptom domains (Table 3). Strong correlations were noted for pyramidal system dysfunction (muscle power, spasticity), in agreement with previously reported strong correlations between the Neuro-QOL lower extremity score and the timed 25 foot walk [23]. Only weak to moderate correlations were noted for visual, sensory, cerebellar and cognitive dysfunction, consistent with previous studies with similar analyses [39, 40]. Discrepancy between patients and physicians has many potential reasons. Pain is an inherently subjective experience, which is only partially captured by deficits in the sensory examination. Subjective cognitive complaints are known to be inflated among patients with depression and fatigue [41, 42], while ignored by those with frank dementia, who lose insight into their cognitive status [43]. The visual system score relies mainly upon visual acuity, while the patient may refer to visual impairment as a result of nystagmus, visual field defects and diplopia. Nevertheless, significant correlations between bodily function PROs and quantified neurological examination add credence to the concept of disease monitoring by using PROs.

The lack of perfect agreement between PROs and objective assessments highlight the importance of PROs to get a full picture of the impact of symptoms on QoL [44] as well as for comprehensive evaluation of response to therapy [45]. Indeed, bodily function PROs were independently associated with QoL, while EDSS did not (Table 4), confirming previous reports [46]. Furthermore, it seems that the exact composition of the PROs tool determines the degree of its correlation with objective assessments. In this study, the sum of bodily function PROs was strongly correlated with EDSS (r = 0.77), while the generic QoL questionnaire (WHOQOL-BREF) was only moderately associated with EDSS (r = -0.48). This is not surprising given that our intention in selecting the composition of PROs to be computed into the e-diary was to create a tool that could specifically grade MS symptoms in a comprehensive manner, with emphasis on symptoms frequency and intensity.

An important impediment in using PROs for individual patient clinical decision making is the large fluctuations in their scores, despite apparent clinical stability. For example, it has been shown that Multiple Sclerosis Walking Scale-12 (MSWS-12), a PRO of walking, remained stable over 2 years on the group level, although fluctuations on the individual level were found in up to 80% of the patients, above the minimal clinically important difference for that scale, which takes into account measurement standard errors and test-retest reliability. These fluctuations in walking PROs on the individual level, but not on the group level, were noted despite being free from clinical relapses [47]. In this study, the e-diary derived bodily function PRO (eBF score) was predictive of clinical disease activity on the group level. This summary score increased pre-relapse, decreased post-relapse and was close zero for participants without a relapse (Fig 4). Individual regression slopes moderate random fluctuations in PROs and may be more reliably indicative of change than comparing the last PRO value to the previous one. The small number of relapses in this study (13 events) precluded determination of sensitivity and specificity of individual regression slopes as relapse predictors on the individual level. Mental PROs: depression, anxiety, physical and mental fatigue–are not part of routine relapse determination in our medical center. Therefore, summary score of mental functions was deemed less suitable for the predictive validity analysis.

The maximal change in eBF score was independently associated with both clinical disease activity and the psychological status of patients (Table 5). It follows that change in bodily function PROs was driven by MS relapses, but the degree of change was also influenced by depression and anxiety. This finding is in agreement with previous reports [48]. Both depression [49] and anxiety [50] have been identified as independent predictors of self-rated disability and QoL among patients with MS. Further work is needed to determine thresholds for change in eBF scores or eBF individual regression slopes that could reliably predict clinical disease activity on the individual level. Higher thresholds are expected for patients with clinically significant depression or anxiety.

This study has several limitations. MRI was not part of this research protocol. Only 15 patients had a scan at baseline and by the end of the 1year clinical follow-up, which precluded the integration of MRI into the definition of disease activity in this study. Since the e-diary was considered experimental, patients knew that the information was not consistently used for providing medical care. It is reasonable to premise that if patients knew that the e-diary derived information was affecting their care, adherence to using the e-diary could have been even higher. Due to convenience sample of small size, our findings should be regarded as preliminary. Despite statistically significant difference between pre-relapse slopes and whole-study slopes of change in eBF among participants who did not relapse, the possibility of non-representative sample exists. Therefore, further studies with larger samples are needed to confirm validity of this e-diary, in terms of relapse prediction. Indeed, fully remote mobile health studies have enrolled in the tens of thousands from across distributed geographic regions, providing the opportunity for broad sampling across diverse populations in the real-world setting [51]. It is also unknown if e-diary derived PROs could predict the more subtle and gradual progression, which is independent of relapse activity.

## Conclusion

PwMS seem to adhere to longitudinal PROs collection by using a smartphone-based e-diary application. The construct validity and responsiveness of e-diary derived PROs to change in objective clinical status on the group level, carry the potential for using smartphone-based PRO diaries in clinical research, to monitor therapeutic effects of medications from the patients’ perspective. Further work with a larger samples is needed to determine thresholds for significant change in PROs scores on the individual level, which may alert clinicians about possible disease worsening in between clinic visits. Integration of such PROs with the treating clinician’s perspective may provide a basis for informed and shared therapeutic decisions and may represent a further step in the implementation of digital health in MS care.

## Supporting information

S1 Table Patient reported outcome measures that were periodically collected by the e-diary. (DOCX)

S1 File Sample size considerations. (DOCX)

S2 File De-identified data set. (XLSX)
