# Supplementary material for: Mobile-phone-based e-diary derived patient reported outcomes: Association with clinical disease activity, psychological status and quality of life of patients with multiple sclerosis
Source: PLoS One. 2021 May 5;16(5):e0250647. doi: 10.1371/journal.pone.0250647 (PMC8099126; doi:10.1371/journal.pone.0250647)
Supplement: S1 Table — (DOCX) [file pone.0250647.s001.docx]

**S1 Table: Patient reported outcome measures that were periodically collected by the e-diary**

| **Bodily functions component** | |
| --- | --- |
| ***Patient reported outcome*** | ***Purpose and available evidence for internal consistency, reliability and validity*** |
| Impact of visual impairment scale  (5 items). | **The extent to which various activities dependent upon vision are affected by multiple sclerosis related visual problems.**  Cronbach alpha=0.86 [1]. Strong correlations with other patient reported visual scales [2]. Moderate correlations with 'neurostatus' visual system score (this study). |
| Neuro QOL- lower extremity functions  (8 items) | **The degree of mobility difficulties.**  Cronbach alpha=0.93[3]. Test –retest reliability = 0.91[3]. Strong correlation with timed 25 foot walk [3]. Strong correlations with 'neurostatus' pyramidal system score (this study). Moderate correlation with 'neurostatus' cerebellar system score (this study). |
| Neuro QOL- upper extremity functions  (8 items) | **The degree of difficulty in carrying out fine motor tasks and upper limb activities of daily living.**  Cronbach alpha=0.86[3]. Test –retest reliability = 0.81[3]. Moderate correlation with the 9 hole peg test [3]. Strong correlations with 'neurostatus' pyramidal system score (this study). Moderate correlation with 'neurostatus' cerebellar system score (this study). |
| Pain effects scale  (6 items) | **The degree to which pain and unpleasant sensations interfere with mood, ability to move, sleep, work and enjoyment of life.**  Cronbach alpha=0.86 [4]. Strong correlations with other patient reported sensory scale [2]. Moderate correlation with 'neurostatus' sensory system score (this study). |
| Bladder control scale  (4 items) | **The frequency of bladder control events and their impact on overall lifestyle**.  Cronbach alpha=0.82 [4]. |
| Bowel control scale  (5 items) | **The frequency of bowel control events and their impact on overall lifestyle.**  Cronbach alpha=0.78 [4]. |
| Pseudobulbar affect  (7 items) | **Perceived frequency of pseudo-bulbar affect episodes**[5]**.** Cronbach alpha=0.87; Test-retest reliability =0.88; Sensitivity of 84% and specificity of 81% in predicting neurologists' diagnosis of affective lability [5]. |
| Spasticity  (1 item) | **0—10 numeric rating scale to measure of spasticity**.  Test-retest reliability =0.83; Strong correlation with other spasticity scales [6]. Strong correlations with 'neurostatus' pyramidal system score (this study). |
| **Mental component** | |
| Abbreviated perceived deficits questionnaire  (5 items) | **Subjective self report of cognitive function.**  Cronbach alpha=0.84 [4]. Subjective impairment by this questionnaire predicted objective impairment [7]. Moderate correlation with SDMT-Symbol digit modalities test (this study). |
| Mental health inventory (MHI)  (18 items) | **Degree of anxiety, depression, positive affect and behavioral control.**  Cronbach alpha=0.8- 0.93 for the various constructs [4]. Strong correlations between MHI-anxiety and HADS^a^-anxiety as well as between MHI-depression and HADS^a^-depression (this study). |
| Abbreviated modified fatigue impact scale  (5 items) | **Perceived impact of fatigue on a variety of daily activities.**  Cronbach alpha=0.8 [4]. Strong correlation with health related quality of life among people with multiple sclerosis (this study). |
| Neuro QOL- sleep  (8 itmes) | **Frequency of sleep disturbance**.  Cronbach alpha= 0.81; Test-retest reliability=0.8; Strong correlation with health related quality of life among people with multiple sclerosis [3]. |

1. HADS = Hospital anxiety and depression scale [8]

# References

1. Ritvo PG, Fischer JS, Miller DM, Andrews H, Paty DW, LaRocca NG. Multiple Sclerosis Quality of Life Inventory: A user’s manual. Mult Scler. 1997; 1–65.

2. Marrie R a, Goldman M. Validity of performance scales for disability assessment in multiple sclerosis. Mult Scler. 2007;13: 1176–1182. doi:10.1177/1352458507078388

3. Miller DM, Bethoux F, Victorson D, Nowinski CJ, Buono S, Lai JS, et al. Validating Neuro-QoL short forms and targeted scales with people who have multiple sclerosis. Mult Scler J. 2016;22: 830–841. doi:10.1177/1352458515599450

4. Rivto PG, Fischer JS, Miller DM, Andrews H, Paty DW, LaRocca NG. Multiple Sclerosis Quality of Life inventory: A user’s manual. New York; 1997.

5. Moore SR, Gresham LS, Bromberg MB, Kasarkis EJ, Smith RA. A self report measure of affective lability. J Neurol Neurosurg Psychiatry. 1997;63: 89–93.

6. Farrar JT, Troxel AB, Stott C, Duncombe P, Jensen MP. Validity, reliability, and clinical importance of change in a 0-10 numeric rating scale measure of spasticity: a post hoc analysis of a randomized, double-blind, placebo-controlled trial. Clin Ther. 2008;30: 974–985. doi:10.1016/j.clinthera.2008.05.011

7. Marrie RA, Chelune GJ, Miller DM, Cohen JA. Subjective cognitive complaints relate to mild impairment of cognition in multiple sclerosis. Mult Scler. 2005;11: 69–75. doi:10.1191/1352458505ms1110oa

8. Zigmond AS, Snaith RP. The hospital anxiety and depression scale. Acta Psychiatr Scand. 1983;67: 361–70.
