# Supplementary material for: Mobile-phone-based e-diary derived patient reported outcomes: Association with clinical disease activity, psychological status and quality of life of patients with multiple sclerosis
Source: PLoS One. 2021 May 5;16(5):e0250647. doi: 10.1371/journal.pone.0250647 (PMC8099126; doi:10.1371/journal.pone.0250647)
Supplement: S1 File — (DOCX) [file pone.0250647.s002.docx]

**S1 File: Sample size considerations.**

We aimed at evaluating the construct and predictive validity of e-diary derived patient reported outcomes (PROs).

For construct validity, we planned to correlate e-diary derived PRO scores with quantified neurological examination (according to 'neurostatus' functional system scores and Symbol Digit Modalities Test).

We calculated the sample size to have a power of 80% at a confidence level of 0.05 to test correlation coefficient difference from zero, assuming a correlation coefficient of at least 0.3. A sample size of 95 patients was required after inflation to compensate for loss of 10% of recruited subjects.

For predictive validity, we planned to compare pre-relapse regression slopes of e-diary derived sum of bodily function (eBF) scores with whole study regression slopes of patients without clinical MS relapses. Based on previous data on relapse rate among people with multiple sclerosis treated with DMTs [1], we estimated that about 20% of recruited patients would experience a relapse during the study year.

We calculated the sample size to have a power of 80% at a confidence level of 0.05 to detect a mean difference of 1 ± 0.5 point per month between pre-relapse regression slopes of eBF and whole study regression slopes of patients without on-study clinical relapses, which were estimated to be close to zero (0 ± 0.5).

A sample size of 63 patients was required after inflation to compensate for loss of 30% of recruited subjects. We considered greater degree of loss to follow-up for the predictive validity analysis, as it required multiple reports over few months, rather than a single report at baseline, that was sufficient for the purpose of construct validity estimation.

Sample size estimations were carried out using Winpepi, version 11.48 [2].

# References

1. Inusah S, Sormani MP, Cofield SS, Aban IB, Musani SK, Srinivasasainagendra V, et al. Assessing changes in relapse rates in multiple sclerosis. Mult Scler. 2010;16: 1414–1421. doi:10.1177/1352458510379246

2. Abramson JH. WINPEPI updated: Computer programs for epidemiologists, and their teaching potential. Epidemiologic Perspectives and Innovations. Epidemiol Perspect Innov; 2011. doi:10.1186/1742-5573-8-1
